# Commentary: Progressive multifocal leukoencephalopathy genetic risk variants for pharmacovigilance of immunosuppressant therapies

**DOI:** 10.3389/fneur.2023.1146027

**Published:** 2023-03-16

**Authors:** Joseph R. Berger, Hans-Peter Hartung

**Affiliations:** ^1^Department of Neurology, Perelman School of Medicine, The University of Pennsylvania, Philadelphia, PA, United States; ^2^Department of Neurology, Medical Faculty, Heinrich Heine University, Düsseldorf, Germany; ^3^Brain and Mind Center, University of Sydney, Sydney, NSW, Australia; ^4^Department of Neurology, Palacky University, Olomouc, Czechia

**Keywords:** progressive multifocal leucoencephalopathy (PML), multiple sclerosis, natalizumab, risk factors, immunogenetics

## Introduction

Progressive multifocal leukoencephalopathy (PML) is a dreaded serious complication of immunotherapies. The drug with which it has been most frequently associated is natalizumab, a monoclonal antibody directed to alpha integrin that prevents the entry of lymphocytes to the central nervous system. It is approved for the treatment of relapsing multiple sclerosis (1, 2). As of 31 July 2022, 895 cases (892 in MS and 3 in patients with Crohn's disease) with a global overall incidence of 3.1 per 100,000, with 215 recorded deaths and 690 survivors with varying degrees of disability have been recorded (Biogen data on file). Previously, a number of risk factors were identified for the development of PML in patients on natalizumab: prior exposure to immunosuppressants, the duration of natalizumab treatment, and the presence of antibodies to the causative agent JC virus (human polyomavirus 2, HuPyV-2) (1, 2). There has been an intensive search to determine molecular factors governing susceptibility to this drug-related catastrophic CNS infection. Genetic variations have long been suspected to play an important role.

## Genetic risk factors

In a refinement of an earlier study in which 19 genes were identified as increasing the risk of progressive multifocal leukoencephalopathy (PML) (3), Hatchwell et al. performed a case–control analysis that matched patients with PML to JCV antibody-positive patients with multiple sclerosis on natalizumab for 2 or more years who did not develop PML. This study demonstrated that four gene variants from those with natalizumab-associated PML are robustly linked to the risk of drug-associated PML (4). In total, two of these four genes only appeared in cases of drug-associated PML and were never observed in the drug-exposed controls. None of the drug-exposed PML cases was presented with more than one of the four genetic variants. The presence of any one of the four variants was observed in 10.9% of the drug-exposed PML cases vs. only 1.4% of the drug-exposed controls. When drug-associated patients with PML were compared to drug-exposed matched controls, the risk of PML with any one of these variants was exceptionally high (*p*-value 3.50 E-06, OR = 8.7 (3.7–20.6) (4).

All four genes (LY9, STXBP2, C8B, and FCN2) are involved in immune mechanisms, including viral defense mechanisms. LY9 encodes an immunomodulatory receptor on the surface of T-lymphocytes (5). STXBP2 encodes proteins that are involved in intracellular trafficking and the release of cytotoxic granules by natural killer cells and is associated with familial hemophagocytic lymphohistiocytosis (6). The other two genes (C8B and FCN2) are involved in complement activation. C8B codes for the late-acting complement proteins (C5-C9) that form the membrane attack complex (7), whereas, FCN2 is involved in the lectin pathway of complement (8).

## Genetic risk factors related to host anti-pathogen defense mechanisms

These findings are not surprising. The immune system controls the response to infectious disease, conferring either vulnerability or resistance to a specific pathogen. Host adaptations to infectious pathogens have been among the strongest selective forces on the human genome (9). The expression of illness and its severity are simply the consequence of the combination of the offending organism and the host's response. The latter is determined by immunogenetics. Examples of this interaction are abundant. For instance, the Black Death of the middle ages that resulted from the bacterium *Yersinia pestis* led to an increase in salutary genetic variants in the human population that alters the cytokine response to *Y. pestis* and increase intracellular control of the pathogen in macrophages (10), thereby providing resistance to infection. CCR5-Δ32 deletion that confers resistance to HIV-1 infection has been attributed to this plague (11). In contrast, there are examples in which disease susceptibility is increased, such as deficiency of the membrane attack complex and properdin increasing the invasive nature of *Neisseria* infection; IL-12/23 and interferon-gamma deficiencies increasing the likelihood of disseminated tuberculosis; and signaling lymphocytic activation molecule (SLAM)-associated protein deficiency increasing the risk of X-linked lymphoproliferative disease with Epstein–Barr virus (12). More recently, the genetic variants of cytokine genes have been associated with COVID-19 disease susceptibility and cytokine storm (13). With respect to the genes identified in this study, the polymorphisms of FCN2 increase the risk of recurrent and severe streptococcal infections and rheumatic heart disease (14). Therefore, it should not be surprising that certain immune gene variants involved in the response to the JC virus (human polyomavirus 2) enhance the risk of PML.

## Four PML epochs and risk factors

When considering the underlying risk factors for PML, four epochs of PML are identified. The first epoch was encompassed from the time of its framing as an illness in 1958 by Astrom et al. (15) to the beginning of the AIDS era in 1981. During this timeframe, the disease was associated chiefly, albeit not exclusively, with hematological malignancies, particularly B cell disorders (16). The second epoch began with the AIDS pandemic. PML was observed to be remarkably common with HIV infection ultimately occurring in 4–10% of patients with AIDS (17). HIV/AIDS became and remains the single greatest predisposing factor for PML. The third epoch of PML occurred with the introduction of highly active antiretroviral therapies in 1996 when the incidence of the disease in HIV/AIDS declined precipitously and almost invariably fatal disease is survived by about 50% of those with AIDS-associated PML (17). The next and most recent epoch of PML was initiated in 2005 with the observation of PML occurring with natalizumab (1, 18–20). While immunosuppressive agents had previously been linked to PML, their use in most individuals was for diseases that also increased the likelihood of PML. However, natalizumab was unique as it was not broadly immunosuppressive. Its α4β1 component prevented lymphocyte interaction with VCAM inhibiting lymphocyte entry into the brain and its α4β7 component interfered with lymphocyte binding to the gut endothelial cells through MAdCAM. Therefore, it is effective against MS and inflammatory bowel diseases. The former mode of action is believed to be responsible for the increased risk of PML with its use. Agents that inhibit α4β7 exclusively, such as vedolizumab, have a vanishingly small risk of PML, if any at all.

## Risk mitigation

Although HIV/AIDS remains the most common predisposing cause of PML, a substantial and increasing number of patients are exposed to drugs that increase the risk of PML. As the authors highlight, a large number of drugs have been associated with PML risk, and eight drugs currently carry FDA black box warnings. These drugs have varied indications. While risk stratification methods have been developed for natalizumab-associated PML, this is not available for other agents that predispose to PML and would be very difficult to devise for a variety of reasons, including the relatively small numbers of patients who develop PML with some of these drugs and the difficulty excluding the contribution of the underlying disorder or concomitant therapies to the development of PML. Furthermore, despite the broader adoption of the risk mitigation strategy employed with natalizumab, PML remains a substantial concern given the mortality, persistent disability, and the lack of established treatments.

## Discussion

Testing for these genes will not eliminate the risk of PML but can be very helpful in identifying a subpopulation (~10%) at particularly high risk for its occurrence when being treated with drugs that predispose to the disorder. The availability of a simple, relatively inexpensive test that can identify the genes that put one at risk for PML would be enormously helpful in the management of patients. The widespread use of such testing could potentially allow the physician to use alternative therapies that do not carry the same risk of PML, such as using alternative therapies for MS rather than using natalizumab in the JCV-positive individual. In those instances where alternative therapies do not exist, it would alert the treating physician to the importance of careful and frequent evaluation of PML. Tests for these genes would also be helpful for informing the patient and the family about relative risks.

## Author contributions

All authors listed have made a substantial, direct, and intellectual contribution to the work and approved it for publication.
